# LettuceMOT: A dataset of lettuce detection and tracking with re-identification of re-occurred plants for agricultural robots

**DOI:** 10.3389/fpls.2022.1047356

**Published:** 2022-11-18

**Authors:** Nan Hu, Shuo Wang, Xuechang Wang, Yu Cai, Daobilige Su, Purevdorj Nyamsuren, Yongliang Qiao, Yu Jiang, Bo Hai, Hang Wei

**Affiliations:** ^1^ College of Engineering, China Agricultural University, Beijing, China; ^2^ School of Mechanical Engineering and Transportation, Mongolian University of Science and Technology, Ulaanbaatar, Mongolia; ^3^ Australian Centre for Field Robotics (ACFR), The University of Sydney, Sydney, NSW, Australia; ^4^ Horticulture Section, School of Integrative Plant Science, Cornell University, Geneva, NY, United States; ^5^ College of Science, Inner Mongolia Agricultural University, Hohhot, China; ^6^ College of Chemistry and Chemical Engineering, Inner Mongolia University, Hohhot, China

**Keywords:** dataset, agriculture, robotics, deep learning, MOT, lettuce, detection, tracking

## Introduction

With the development of robotics and artificial intelligence, various intelligent robots are being developed and deployed for different autonomous operations in agriculture. Specifically for robotic precision spray of pesticide and fertilizer, modern agricultural robots use various onboard cameras to detect and track various plants on farms. Accurate detection and reliable tracking of plants are of utmost importance for robots to carry out pinpoint spray action for delivering the chemicals to the relevant parts of plants. With the rapid development of artificial intelligence, specially with deep learning methods, robots are able to accomplish detection and tracking of plants using intelligent machine learning algorithms. However, the prerequisite of it is the large amount of labelled data to train, validate and test the machine learning models.

In recent years, many researchers have proposed various datasets to detect and localize fruits and vegetables (Jiang et al., 2019; Moreira et al., 2022). Bargoti and Underwood (2017) presented a fruit detection dataset collected by a robot travelling in fruit orchards. Images in the dataset include mangoes, almonds and apples. Koirala et al. (2019) provided a image dataset which was captured in a mango orchard. At the same time, they also presented a benchmark called ‘MangoYOLO’. Kusumam et al. (2017) collected three-dimensional point cloud data and RGB images of broccoli with their robots in Britain and Spain. Chebrolu et al. (2017) presented a large-scale agricultural dataset of sugar beet, which was collected by their robot traveling in a sugar beet farm near Bonn in Germany. The dataset contains approximately ten thousand images of plants and weeds labelled pixel by pixel, which can be used for semantic segmentation of crop and weed. Bender et al. (2020) utilized the agricultural robot Ladybird to collect a multimodal data including stereo RGB and hyperspectral images of crops and weeds, as well as the environment information. The dataset contains information of cauliflower and broccoli recorded over teen weeks. These datasets have made a significant contribution in the field of plant detection, localization and segmentation (Lottes et al., 2017; Milioto et al., 2018). However, these datasets cannot be directly used for tracking plants, due to the lack of correspondence of plants between consecutive images. Without tracking plants, robots cannot recognize the same plant which shows up in two consecutive images, and might spray it more than once.

More recently, Jong et al. (2022) presented APPLE MOTS, a dataset collected by a UAV and a wearable sensor platform which includes about 86000 manually annotated apple masks for detecting, segmenting and tracking homogeneous objects. They evaluated different MOTS (Voigtlaender et al., 2019; Xu et al., 2020) methods on the dataset, and provided benchmark values of apple tracking performance. This dataset can be used for detection, segmentation and tracking of apples. The trajectory of camera mostly follows a simple forward motion, and targets which have gone out of camera field of view do not re-appear again in the following images. However, for robotic precision spray application, robots might reverse back to avoid dynamic obstacles such as a working human. It might also temporarily move out of the row, *e.g.* to refill its battery, and return back to continue its spray work. In such cases, it will unavoidably encounter situations in which plants that have been previously observed and gone out of camera field of view re-appear again. The robots need to re-identify these plants and correlate them again with the same ones previously observed in order to spray each plant exactly once. Otherwise if these plants are identified as new plants, they will be sprayed more than once.

**Figure 1:**
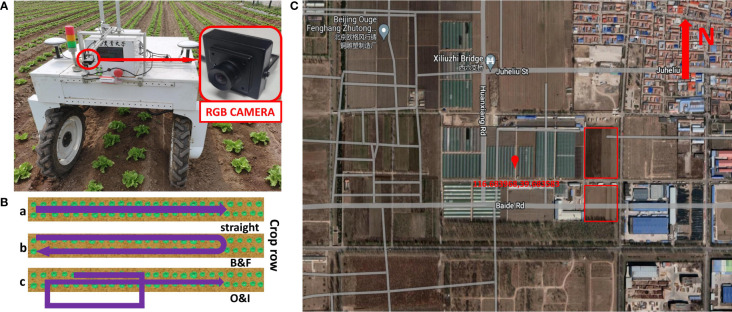
Details of data acquisition. **(A)** A RGB camera was installed in front of the VegeBot, an agricultural robot developed by China Agricultural University, and collected data in lettuce farm. **(B)** Three types of robot motions during image acquisition process. a, b and c correspond to straight, backward & forward, and turning out & turning in robot motions, respectively. **(C)** The location of the farm on satellite image. The red box is the place where the image is collected.

The main contributions of the proposed dataset are as follow:

1. A dataset captured by the front downward facing camera of the VegeBot, an agricultural robot developed by China Agricultural University, in two growth stages of lettuce for joint plant detection and tracking research, is provided as shown in **Figure 1**. It contains around 5400 RGB images and their corresponding annotations in the widely used MOT format (Leal-Taixé et al., 2015; Milan et al., 2016; Dendorfer et al., 2020; Dendorfer et al., 2021). The dataset is publicly available at: https://mega.nz/folder/LlgByZ6Z#wmLa-TQ8NYGkPrJjJ5BfQw.
2. The proposed dataset fills the current shortage of plant detection and tracking agricultural dataset in which the challenging situation of re-identification of re-occurred plants exists. By tackling such a challenging problem, the agricultural robot ensures to spray each plant exactly once even if it observes the same plant more than once during back and forth motion, or it re-enters the same row in the farm. Four state-of-the-art MOT methods are tested on the proposed dataset, and the benchmark results are reported for comparison.

## Materials and methods

### Data acquisition

The dataset in this paper were collected in a lettuce farm in Tongzhou District, Beijing, China in April and May 2022. The images of lettuce are captured during its two growth stages, which are namely rosette stage and heading stage, respectively. The distance between two adjacent lettuces in the same row is approximately 0.3-0.35 m, and the distance between two rows of lettuce is about 0.3 m. Due to regular weeding, the maximum weed density is 10 per square meter.

The images were collected by VegeBot when it traveled through different rows of the farm. VegeBot is a four-wheel-steer and four-wheel-drive agricultural robot developed by China Agricultural University, which is specifically designed for autonomous operations in vegetable farms. Key parameters of the robot are summarized in **Table 1**. The VegeBot is equipped with a RTK-GPS sensor for GNSS based global localization, a front downward facing USB RGB camera for the perception of vegetable plants, and a downward facing Intel RealSense D435i depth camera with IMU sensor for collecting additional depth and heading angle information. During the data collection process of this paper, only the front downward facing USB RGB camera was utilized. When collecting data, in order to ensure the quality of the collected data, the vision based autonomous navigation functionality of the robot was disabled, and it was manually driven with a remote controller on the farm. Since the velocities and steering angles of four wheels can be independently controlled, the motion of the robot can adopt different styles. When the robot traveled straight along the farm lane, it adopted the Ackerman motion style for smooth forward or backward motion with smaller angular velocity. When it turned and switched to another lane, it adopted four-wheel-steer motion to ensure a large rotation angle.

**Table 1:** Key parameters of VegeBot.

Parameter	Value
Length	1.2 m
Width	1.1 m
Height	1.1 m
Weight	approx. 350 kg
Max Load	200 kg
Max Speed	1.2 m/s

As shown in **Figure 1**, images are acquired by a RGB camera, which is installed approximately 1.5 m away from the ground. The model of camera is Vishinsgae SY011HD. It is equipped with a 1/2.7-inch AR0230CS digital image sensor, and the pixel size of it is 3 µm × 3 µm. The maximum resolution and maximum frame rate of the camera are 1920×1080 and 30 FPS, respectively. The manufacturer of the camera lens is Vishinsgae, and the f-number of 2.8 is utilized. The wide-angle of the camera lens is 130 degree. Images are cropped into the resolution of 810×1080 to remove unrelated area for better detection and tracking. The camera exposure time is automatically set by the camera. The images are captured under natural ambient light condition, and they are extracted from the original video format. The camera acquires images at the frequency of 10 FPS, with the average overlap between two consecutive images larger than 2/3 of the image.

During the data acquisition process, the robot follows three types of motions, *i.e.* straight, backward and forward, and turning out and turning in, as shown in **Figure 1B**. Among them, the data recorded with the robot motions backward and forward (denoted as B&F) and turning out and turning in (denoted as O&I) contains re-occurrence of lettuce plants after having gone out of the camera field of view. In comparison, plants will not re-appear again after having gone out of the camera field of view in the data recorded with the robot straight motion (denoted as straight). The speed of the robot is between 0.3 m/s to 0.4 m/s when it travels straight forward or backward, and between 0.1 m/s to 0.2 m/s when it turns.

### Image annotation and dataset construction

**Figure 2:**
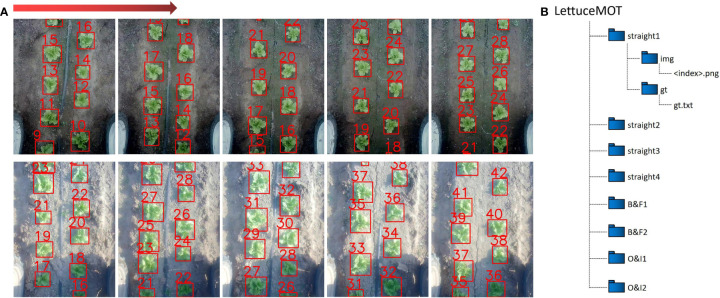
Details of the dataset. **(A)** Sample images and their corresponding annotations. The direction of the red arrow indicates the order of images. **(B)** The file structure of the dataset.

The image labeling and annotation tool *DarkLabel^1^* is utilized to label the collected images. The images are labeled with the MOT format (Leal-Taixé et al., 2015; Milan et al., 2016; Dendorfer et al., 2020; Dendorfer et al., 2021), which is denoted as follow,


(1) $$label=\left\{frame_{id},id,x,y,w,h,1,1,1\right\}$$

where *frame_id_* is the ID number of the frame, *id* is the ID number of the target, *x* and *y* are the coordinates of the upper left corner of the annotated box, and *w a*nd *h* represent the width and height of the box. The last three numbers in the label format are not used in our dataset, and therefore they are all filled with ones. A sample image and its corresponding annotation are shown in **Figure 2A**. The dataset consists of eight parts, with each growth stage containing four parts, and contains 5466 RGB images in total. Each growth stage consists of two straight parts, one B&F part and one O&I part. There are 2745 RGB images in the rosette stage and 2721 RGB images in the heading stage. The details of the dataset are summarized in **Table 2**. Wherein, Tracks and Boxes refer to the total number of objects and the total number of bounding boxes in an image sequence, respectively.

**Table 2:** Summary of eight parts of the dataset.

Dataset	*Straight*1^a^	*Straight*2^a^	*B*&*F*1^b^	*O*&*I*1^c^	*Straight*3^a^	*Straight*4^a^	*B*&*F*2^b^	*O*&*I*2^c^
Resolution(Pixel)	810×1080	810×1080	810×1080	810×1080	810×1080	810×1080	810×1080	810×1080
Length(Frame)	534	550	540	1121	525	402	612	1182
Tracks(Track)	95	122	52	43	133	106	81	75
Boxes(Box)	4745	4960	4547	6510	4944	4164	5857	7008
The growth stage	The rosette stage				The heading stage			
The weather	Cloudy				Sunny			
Light intensity	Weak				Strong			
Acquisition time	Afternoon, April 25th, 2022				Afternoon, May 2nd, 2022			

a The set straight is the images collected by the robot traveling straight from the starting point to the end point.

b The set B&F is collected when the robot travels straight to the end point, and then reverses back to the starting point.

c The set O&I is collected when the robot travels straight, turns out of the current row and travels back, and finally returns to the the same row and keeps moving forward.

The structure of the dataset is shown in **Figure 2B**. The eight parts of dataset are stored in eight folders, each of which contains two folders, *i.e. img* and *gt*. The images captured by the robot are contained in the *img* folder, whose corresponding annotations are contained in the *gt.txt* file in the *gt* folder. All image files are named by their numerically orders and are in *PNG* format.

Four state-of-the-art MOT methods are tested on the proposed dataset, which are ByteTrack (Zhang et al., 2021a), ByteTrack with NSA Kalman filter (Du et al., 2021), FairMOT (Zhang et al., 2021b), and SORT (Bewley et al., 2016)^2^. They are finetuned on our dataset using their default hyperparameters. The results are summarized in **Table 3**. *straight2* and *straight4* are used to train each model and inference is performed on the other six parts. All training and testing are carried out on a NVIDIA RTX 2080Ti GPU. It can be seen from **Table 3** that ByteTrack with NSA Kalman filter performs better than the other three methods on the test sets. FairMOT performs poorly when objects are very similar due to the application of appearance features. Therefore, it is easier to obtain better results by using motion features on our dataset. However, due to the lack of the ability to re-identify re-occurred objects in these methods, the performance metrics, e.g. the IDSW, on B&F and O&I are generally poor, where lettuces go out of the camera field of view and re-occur later. To tackle such a challenging tracking problem, successful MOT methods can potentially try to extract unique feature of each lettuce plant, e.g. the color feature of its surrounding soil or the graph structure of the target lettuce with its neighbors. By matching the composed unique feature, a successful MOT method should search from all targets in history, and find out the correct targets for the re-occurred objects.

**Table 3:** Performance of four MOT methods with the proposed LettuceMOT.

Dataset	Method	MOTA(%)↑^a^	HOTA(%)↑	DetA(%)↑	AssA(%)↑	IDF1(%)↑	*FPS↑ *	IDSW↓
straight2	YOLOV5+SORT	86.512	76.759	76.030	77.548	92.907	**96.50**	**1**
	FairMOT	82.097	73.120	72.921	73.852	88.715	28.10	85
	ByteTrack	70.323	61.440	58.603	65.091	82.898	30.06	2
	ByteTrack+NSA kalman filter	**90.202**	**87.238**	**86.310**	**88.185**	**94.718**	29.48	3
B&F1	YOLOV5+SORT	88.344	61.777	80.137	47.665	58.776	**97.75**	43
	FairMOT	83.418	57.633	75.136	44.413	55.945	29.34	84
	ByteTrack	75.500	49.910	62.591	40.152	53.996	29.73	**42**
	ByteTrack+NSA kalman filter	**91.929**	**68.656**	**89.786**	**52.501**	**59.695**	29.27	43
O&I1	YOLOV5+SORT	88.710	60.690	80.618	45.749	56.616	**77.00**	38
	FairMOT	81.843	56.172	74.239	42.785	54.760	30.09	51
	ByteTrack	83.533	55.171	72.352	42.121	55.581	30.09	34
	ByteTrack+NSA kalman filter	**89.908**	**65.612**	**85.808**	**50.200**	**58.726**	29.93	**32**
straight4	YOLOV5+SORT	84.990	75.460	74.550	76.407	92.177	**96.75**	**0**
	FairMOT	66.907	62.879	60.777	65.311	78.856	29.18	49
	ByteTrack	63.929	54.938	51.811	59.125	78.382	29.57	**0**
	ByteTrack+NSA kalman filter	**86.431**	**84.413**	**84.008**	**84.834**	**92.722**	29.47	**0**
B&F2	YOLOV5+SORT	84.514	56.064	74.464	42.261	51.667	**95.14**	72
	FairMOT	63.958	43.124	60.272	31.260	43.150	29.27	252
	ByteTrack	58.904	39.581	48.950	32.754	46.337	30.07	**71**
	ByteTrack+NSA kalman filter	**86.512**	**62.728**	**84.015**	**46.856**	**52.074**	29.74	72
O&I2	YOLOV5+SORT	**80.693**	**55.442**	**71.000**	43.363	**53.572**	**90.31**	54
	FairMOT	74.472	52.734	69.528	40.128	50.855	30.14	61
	ByteTrack	48.673	49.729	57.815	42.829	45.699	29.72	**53**
	ByteTrack+NSA kalman filter	51.299	52.904	61.916	**45.225**	46.254	29.40	**53**

^a^Symbols ↑ and ↓ after the evaluation metrics indicate the value of it is the higher the better or the lower the better, respectively. The bold numbers show the best performing method. MOTA, HOTA, DetA, IDF1 and AssA are comprehensive evaluation metrics for MOT methods. IDSW is the number of the times that IDs of the same targets change during tracking. Details of these metrics can be found in (Bernardin and Stiefelhagen, 2008; Li et al., 2009; Ristani et al., 2016; Luiten et al., 2021).

## Data availability statement

The datasets presented in this study can be found in online repositories. The names of the repository/repositories and accession number(s) can be found below: The direct link to the data is https://mega.nz/folder/LlgByZ6Z\#wmLa-TQ8NYGkPrJjJ5BfQw. The name of the repository is ‘LettuceMOT’.

## Author contributions

DS, PN, YQ, YJ, BH and HW contributed to the design of the data acquisition. NH, SW, XW, and YC collected the experimental data. NH, SW and XW organized and labelled the data. NH, SW and DS wrote the first draft of the manuscript. PN, XW, YQ and YJ wrote sections of the manuscript. All authors contributed to manuscript revision, read, and approved the submitted version.

## Funding

This research was financially supported by the National Natural Science Foundation of China (Grant No. 3217150435), and China Agricultural University with Global Top Agriculture related Universities International Cooperation Seed Fund 2022.

## Acknowledgments

We would like to thank the editor and reviewers for their valuable input, time, and suggestions to improve the quality of the manuscript.

## Conflict of interest

The authors declare that the research was conducted in the absence of any commercial or financial relationships that could be construed as a potential conflict of interest.

## Publisher’s note

All claims expressed in this article are solely those of the authors and do not necessarily represent those of their affiliated organizations, or those of the publisher, the editors and the reviewers. Any product that may be evaluated in this article, or claim that may be made by its manufacturer, is not guaranteed or endorsed by the publisher.

## Footnotes

1 https://github.com/darkpgmr/DarkLabel.

2 The following open source implementations are used in the experiment. ByteTrack: https://github.com/ifzhang/ByteTrack, FairMOT: https://github.com/ifzhang/FairMOT, YOLOV5: https://github.com/ultralytics/yolov5, SORT: https://github.com/abewley/sort and NSA Kalman filter: https://github.com/dyhBUPT/StrongSORT.
